# Atomic physics determination of the fine structure constant

**DOI:** 10.1093/nsr/nwz186

**Published:** 2019-11-15

**Authors:** Zong-Chao Yan

The fine structure constant $\alpha $ is a fundamental physical constant that describes the electromagnetic interaction between charged particles and serves as the coupling constant of quantum electrodynamics (QED). It is dimensionless and thus remains the same under all systems of units. It is worth noting that $\alpha $ cannot be calculated by QED itself; it must be determined experimentally, often with the help of QED directly or indirectly. Determinations of $\alpha $ from different sources can be used to test QED and other sectors of the Standard Model of particle physics, provided both theory and experiment can reach a sufficiently high precision. However, one cannot both test QED and measure α simultaneously, and so at least two independent measurements are required. One of the two most precise determinations of $\alpha $ so far is from the anomalous magnetic moment or ${g_e} - 2$ of the electron, which yields $\alpha $ to an accuracy of 0.24 ppb (parts per billion) [1]. The other one comes from the cesium recoil experiment that gives rise to$\ \alpha $ at 0.20 ppb [2]. The ${g_e} - 2$ determination of $\alpha $ involves a monumental QED calculation, whereas the Cs recoil one relies on QED only in an indirect way, because the Rydberg constant ${R_\infty }\ $used in the Cs recoil determination was already established to very high precision by hydrogen and deuterium transition frequencies together with their corresponding QED calculations. However, these two determinations of $\alpha $ have a 2.5$\sigma $ discrepancy that may have some implications for new physics beyond the Standard Model [2]. From a metrology viewpoint, with the adoption of the 2019 redefinition of the SI base units, a more precise value of $\alpha $ would mean a more precise value of the electron mass$\ {m_e}$ according to$\ \ {R_\infty } = {\alpha ^2}\ {m_e}( {c/4\pi \hbar } )$.

The helium 2^3^*P_J_* (*J* = 0, 1, 2) fine structure is another venue to derive a precise value of$\ \alpha $, as was first proposed by Schwartz [3] in 1964 aiming at a ppm (parts per million) determination. It differs from either the ${g_e} - 2$ or the Cs recoil determination in that it is a *bound-state* QED problem. Compared to the electron ${g_e} - 2$ problem, a splitting between two fine structure levels in 2^3^*P_J_* is more sensitive to $\alpha $ by a factor of ∼137. Helium is also more appealing to experimentalists than hydrogen since the 2^3^*P_J_* energy levels in helium are more widely spaced than 2^2^*P_J_* in hydrogen, and the lifetime of 2^3^*P* is a factor of 100 longer than 2^2^*P*. Historically, the first breakthrough for realizing the Schwartz proposal was the derivation of order ${m_e}{\alpha ^6}$ relativistic and QED corrections by Douglas and Kroll [4] in 1974. In 1995, Yan and Drake [5] evaluated these corrections to a very high precision and thus laid a foundation for pursuing a ppb level determination of$\ \alpha $, instead of the original ppm. Other milestones in theory include the work by Zhang, Yan and Drake [6] in 1996 for the QED corrections of order$\ {m_e}{\alpha ^7}\ln \alpha $, the work by Pachucki [7] in 2006 for$\ {m_e}{\alpha ^7}$ and the extension of$\ \ {m_e}{\alpha ^7}$ to helium-like ions by Pachucki and Yerokhin [8] in 2010.

Since Schwartz's proposal was published, significant progress has been made on the experimental frontier using various measurement techniques. The interplay between theory and experiment has stimulated measurements with ever-increasing accuracy. Some systematic effects, long considered negligible, are now becoming important, such as quantum interference. Recently, the group at University of Science and Technology of China (USTC) led by Shui-Ming Hu [9] has measured the fine structure splittings ${\nu _{02}}$ and$\ {\nu _{12}}$ in the 2^3^*P_J_* manifold of helium by laser spectroscopy and achieved an accuracy of 4 and 80 ppb, respectively. Their results are in good agreement with the QED calculations of Pachucki and Yerokhin [8] up to order$\ {m_e}{\alpha ^7}$. The significance of the work from Hu's group is that, once the theory at the next order ${m_e}{\alpha ^8}$ is completed, an atomic physics value of $\alpha $ at 2 ppb could be derived. Very recently, a microwave measurement of ${\nu _{12}}$ at 10 ppb by Hessels's group at York [10] was reported. Although the York's result is a factor of 8 more precise than the corresponding USTC value, it disagrees not only with the USTC one by about 4.5 standard deviations, but also with the theory [8] by about 1.5 times the theory uncertainty. In order to resolve these discrepancies, more measurements and at least one independent QED calculation are highly desirable. It is expected that the experimental uncertainty in the larger splitting ${\nu _{02}}$ could be reduced to below 1 ppb in the near future [10] and thus an atomic physics value of $\alpha $ at 0.5 ppb or below could be determined, which would be comparable to the ${g_e} - 2$ and Cs recoil values. Before entering this exciting sub-ppb era, however, the most challenging task that remains ahead for theorists is to calculate the QED effect at order$\ {m_e}{\alpha ^8}$.
